# Supplementary material for: Cell-Free Protein Synthesis as a Method to Rapidly Screen Machine Learning-Generated Protease Variants
Source: ACS Synth Biol. 2025 Apr 30;14(5):1710–8. doi: 10.1021/acssynbio.5c00062 (PMC12090339; doi:10.1021/acssynbio.5c00062)
Supplement: Supplementary file 5 — sb5c00062_si_005.pdf [file sb5c00062_si_005.pdf]

Supplementary information: Cell-free protein synthesis as a method to rapidly screen machine learning-directed protease variants

Ella Lucille Thornton (1)*, Jeremy T. Boyle (1), Nadanai Laohakunakorn (1), Lynne Regan (1)*

1. Centre for Engineering Biology, Institute of Quantitative Biology, Biochemistry and Biotechnology, School of Biological Sciences, University of Edinburgh, EH9 3BF, Scotland

*To whom correspondence should be addressed. Email: lynne.regan@ed.ac.uk, ella.thornton@ed.ac.uk

Supplementary Figures:
1. DoE for protease expression
2. Calibration of FRET substrate against known concentrations of protease
3. Identity of all original random mutants
4. Time-course FRET data for 48 B mutants
5. Dataset of FRET data truncated at 0.55
6. Validation of fitness scoring against FRET time-course data
7. Full list of output variants from ALDE
8. Affinity purification SDS-PAGE
9. Size exclusion chromatography SDS-PAGE
10. Purified variants stocked at equimolar concentrations SDS-PAGE

Supplementary tables:
1. Con1 annotated DNA and protein sequence
2. FRET substrate annotated DNA and protein sequence
3. Oligonucleotides used in this study

Supplementary data (provided as individual files):
1. Figure 4 dataset A mutants
2. Figure 4 dataset B mutants
3. ML suggested variant dataset for Figure 5
4. Comparison of CFPS and purified variants for Figure 6

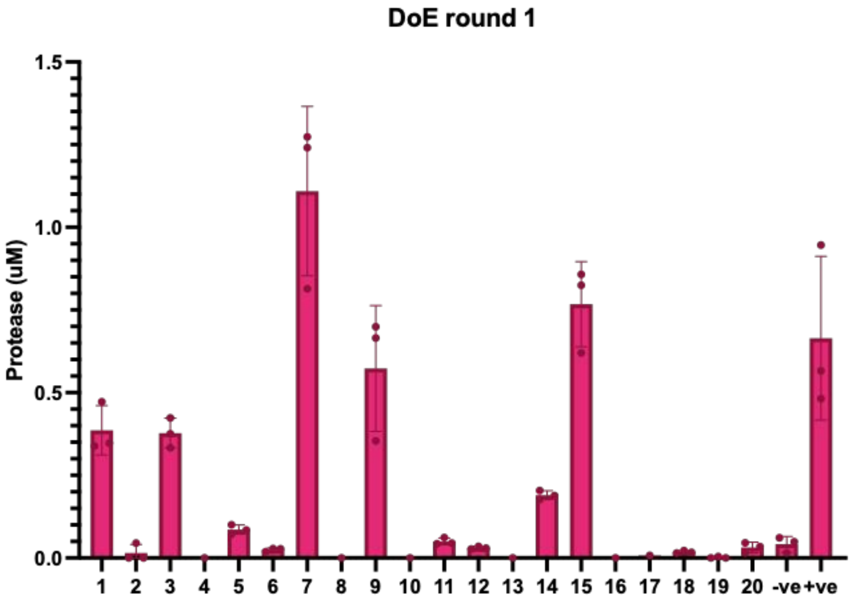

First round:			
Total K (mM)	Total Mg (mM)	PEG (%)	Yield (uM)
110.00	11.00	3	0.39
228.90	26.74	3	0.02
227.46	10.41	5	0.38
107.70	26.46	5	0.00
168.78	18.72	4	0.09
168.78	18.72	4	0.03
229.86	10.97	3	1.11
110.00	27.00	3	0.00
108.66	10.69	5	0.57
226.50	26.18	5	0.00
168.78	18.72	4	0.05
168.78	18.72	4	0.03
94.53	18.89	4	0.00
243.03	18.54	4	0.19
169.38	8.86	4	0.77
168.18	28.58	4	0.00
170.00	19.00	2.75	0.01
167.28	18.37	5.25	0.02
168.78	18.72	4	0.00
168.78	18.72	4	0.03

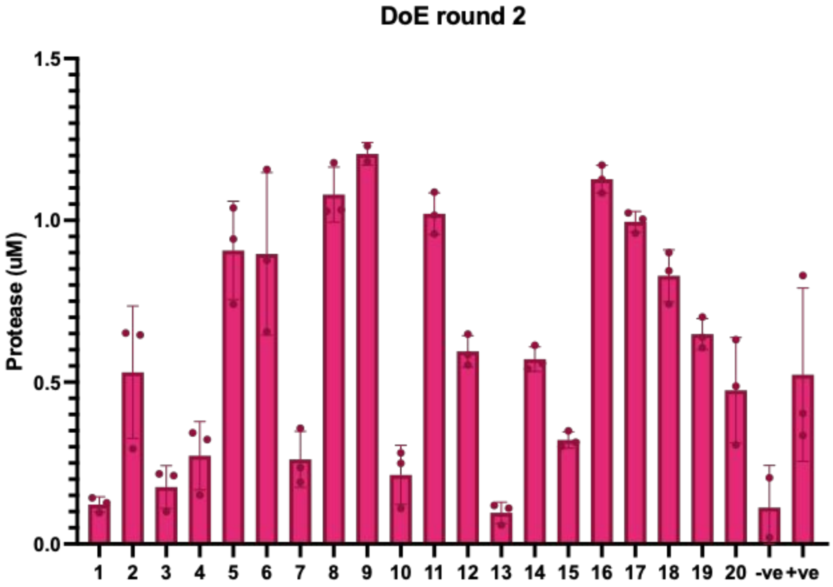

Second round:			
K	Mg	PEG	Yield
220	7.7	1	0.12
300	12	1	0.53
299.36	7.5502	3	0.18
219.901	11.97	3	0.27
270	10.5	2	0.91
270	10.5	2	0.90
300	7.7	1	0.26
220	12	1	1.08
220	7.7	3	1.21
299.1	11.79	3	0.21
270	10.5	2	1.02
270	10.5	2	0.60
207.5	10.5	2	0.10
307.5	10.5	2	0.57
270	7	2	0.32
270	12.375	2	1.13
270	10.5	0.75	1.00
269.1	10.29	3.25	0.83
270	10.5	2	0.65
270	10.5	2	0.47

Figure S1. A Design of Experiments approach to optimise protease expression in CFPS. The experimental design of Central Composite Design was utilised, with axial points. Mg, K, and PEG were selected as variables to assess. The range of concentrations to be tested for each was decided by a literature review. The amount of protease made in each condition was assessed by an activity assay and compared to a purified calibration of protease against FRET substrate (S2). Round one identified conditions that were more favourable for protease expression (as seen by comparison with the +ve sample, with original energy solution components). To further optimise the conditions, another round of DoE was planned and completed, finding a condiiton that increased protease expression from ~0.5 μM to ~1 μM. These conditions were used for all subsequent CFPS experiments.

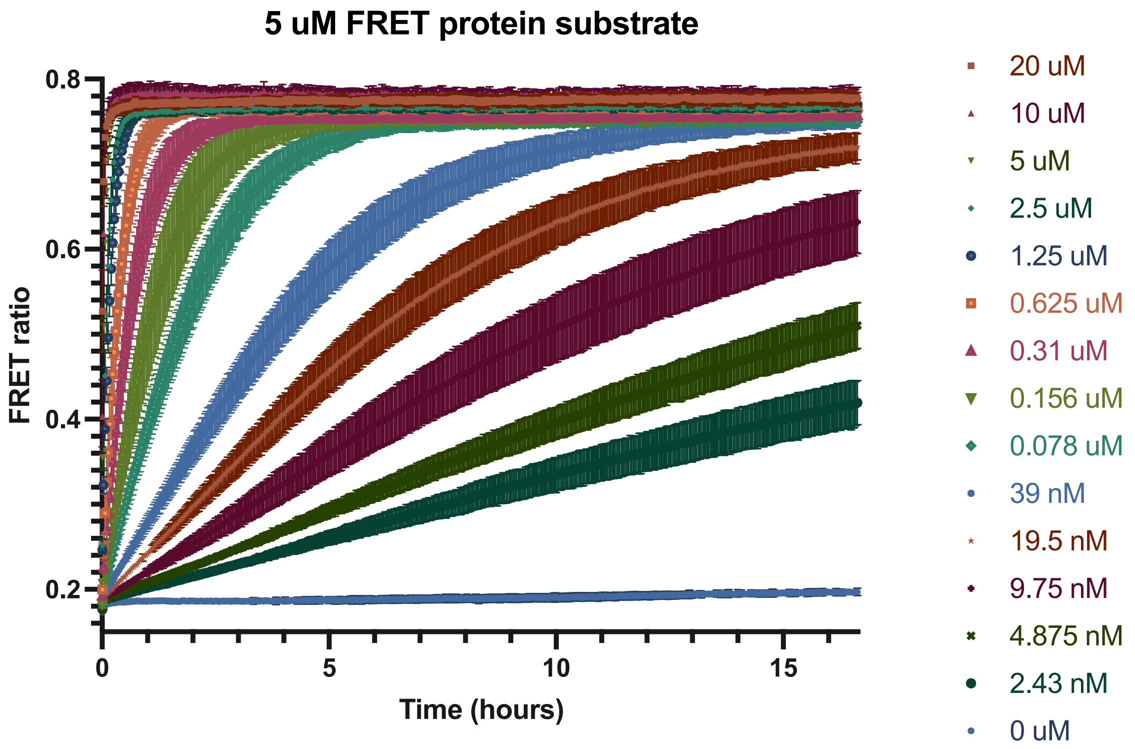

Figure S2. Calibration of FRET substrate against known concentrations of purified Con1 protease. FRET substrate was mixed together with protease in buffer, to give a final FRET concentration of 5 μM. This was immediately measured in a plate reader for fluorescence and subsequent assessment of FRET over time.

Region A:

	Residue:		
Mutant no:	167	169	172
1	E	I	N
2	T	H	T
3	R	M	F
4	L	L	A
5	R	R	L
6	W	A	K
7	R	K	L
8	W	A	F
9	G	F	F
10	Q	L	F
11	H	T	V
12	G	Y	R
13	V	M	N
14	V	T	D
15	Y	F	N
16	W	L	G
17	Q	R	E
18	E	R	W
19	M	S	I
20	D	L	G
21	E	D	W
22	K	S	F
23	K	I	W
24	D	A	G
25	G	S	Y
26	L	T	K
27	R	A	H
28	H	M	E
29	Q	V	T
30	N	K	D
31	E	H	S
32	S	R	F
33	W	R	E
34	A	L	F
35	S	W	S
36	I	E	N
37	I	L	H
38	L	M	D
39	I	A	N
40	L	M	F
41	I	Y	I
42	S	T	E
43	D	D	T
44	E	Q	K
45	N	Q	Y
46	S	D	R
47	Q	L	G
48	K	D	I

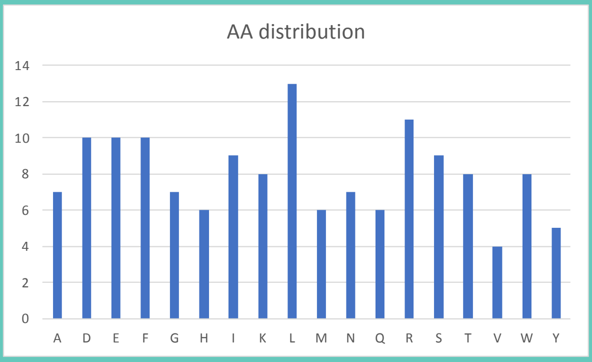

Region B:

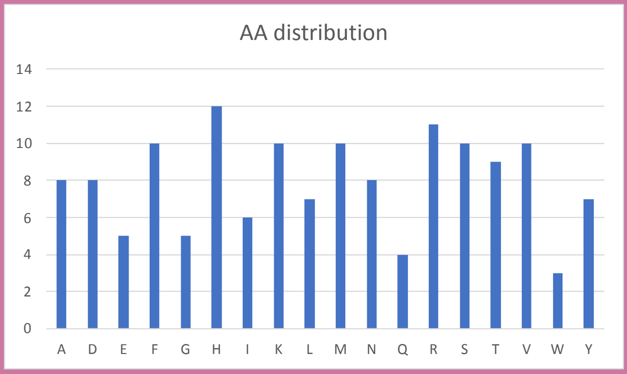

	Residue:		
Mutant no:	217	218	219
1	H	V	L
2	Y	L	I
3	E	G	K
4	N	N	R
5	Q	T	H
6	R	M	H
7	W	A	K
8	H	S	Y
9	H	M	Y
10	L	N	M
11	A	M	G
12	K	F	W
13	F	H	S
14	S	H	F
15	A	V	E
16	M	D	H
17	I	V	T
18	S	K	Q
19	D	H	R
20	R	S	K
21	I	E	D
22	A	R	V
23	W	R	H
24	A	R	F
25	M	D	Y
26	S	V	S
27	G	Y	Y
28	G	N	H
29	F	R	F
30	R	T	A
31	T	K	K
32	F	S	L
33	R	K	N
34	M	T	F
35	Y	G	D
36	K	M	I
37	A	K	T
38	F	L	T
39	Y	V	D
40	F	L	V
41	I	E	N
42	V	A	N
43	M	Q	H
44	I	S	D
45	E	V	T
46	N	Q	V
47	S	D	T
48	M	R	L

Figure S3. Identity of all random mutants. 48 random mutants were created for both region A and region B by a script which compiled a randomised listed of amino acids into a 3 x 48 table. At this stage of the design process, we removed proline and cysteine as possibilities for mutagenesis. The representation of all other amino acids was verified as seen in the bar plots.

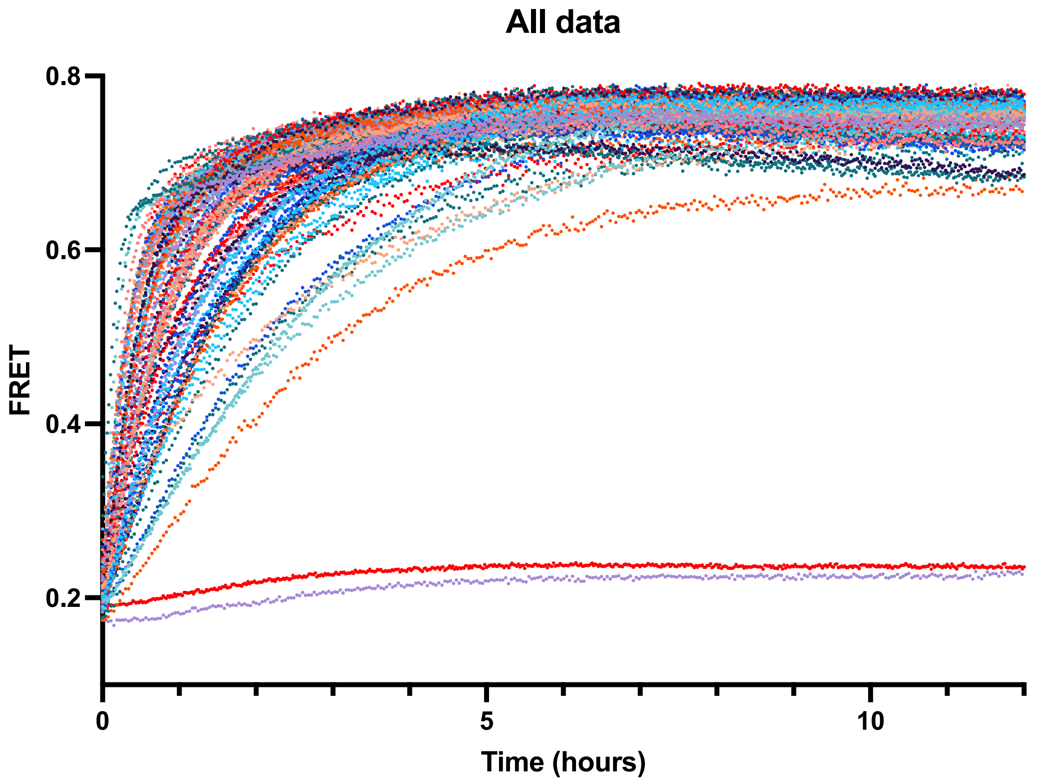

Figure S4. Example time-course data of FRET for 48 B mutants after production by CFPS.

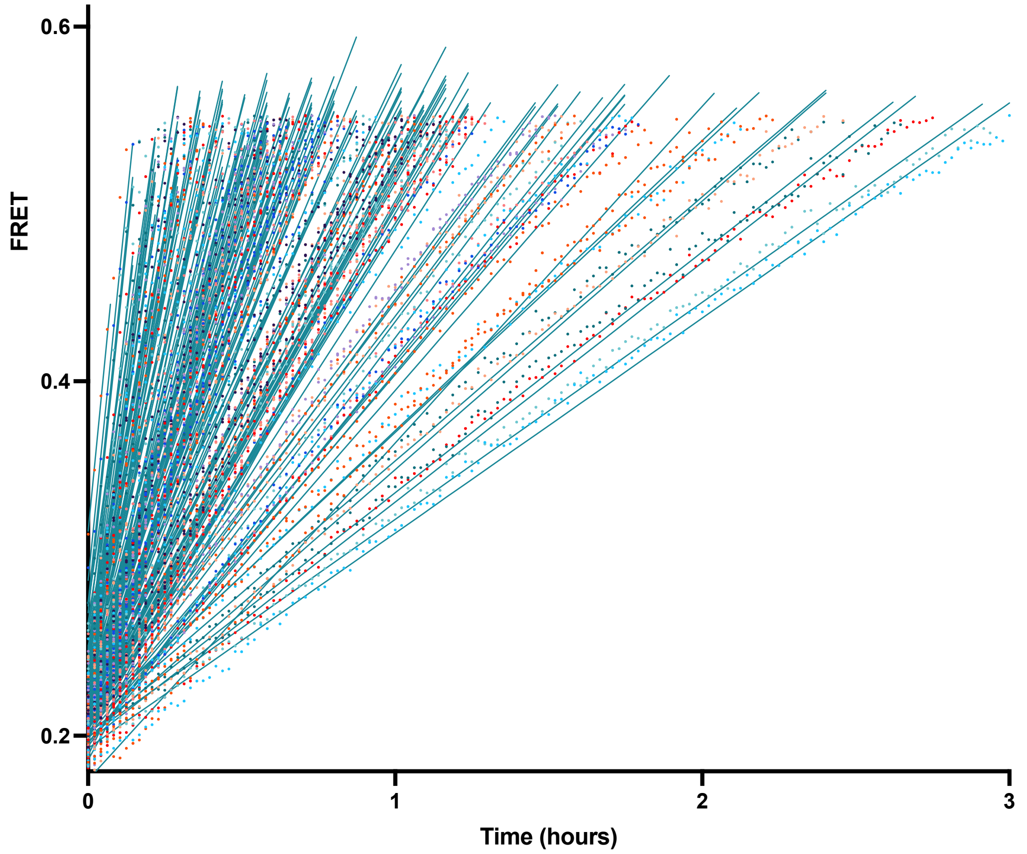

Figure S5. Data from S4 truncated at FRET = 0.55. Plot is cropped to Time = 3 hours to allow closer examination of data points and linear fit. We found this data processing step allowed for accurate scoring of mutant activity, as shown further in Figure S6.

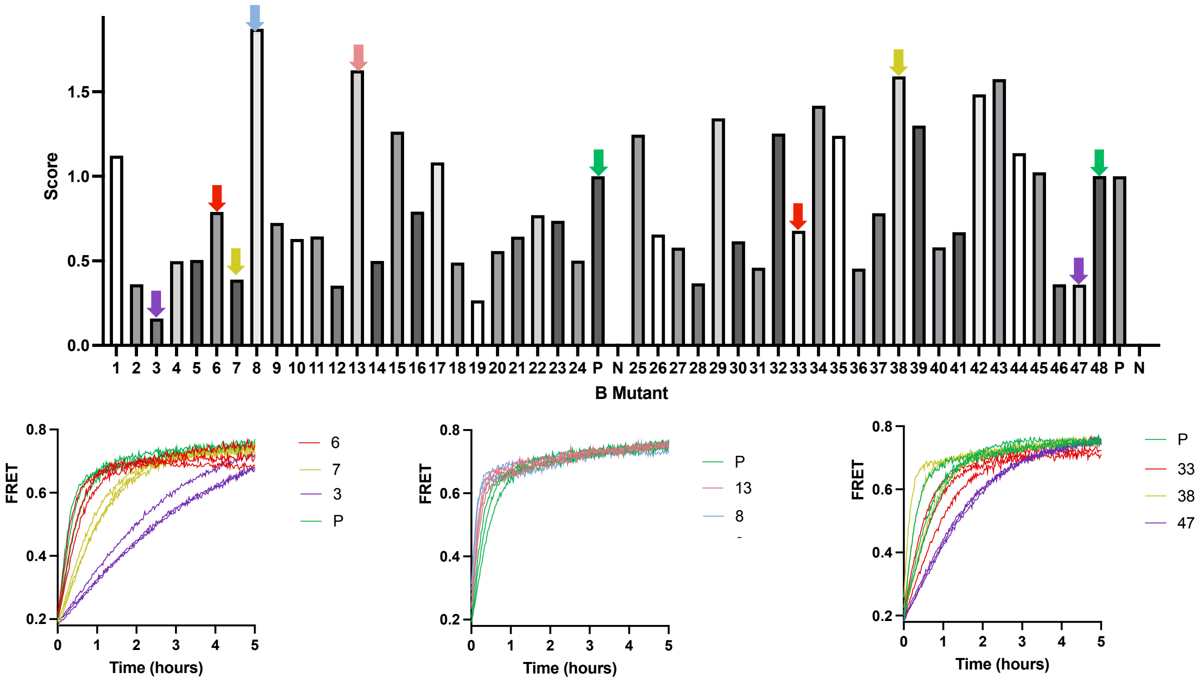

Figure S6. Assessment of final fitness score accuracy against original FRET time-course data. Final scores (average of 3 replicates) are shown in the bar chart for all region B mutants. Below, line charts show individual replicates for selected mutants, the colour of which is then matched to their identity and to the corresponding bar.

Region A: **Region B:**

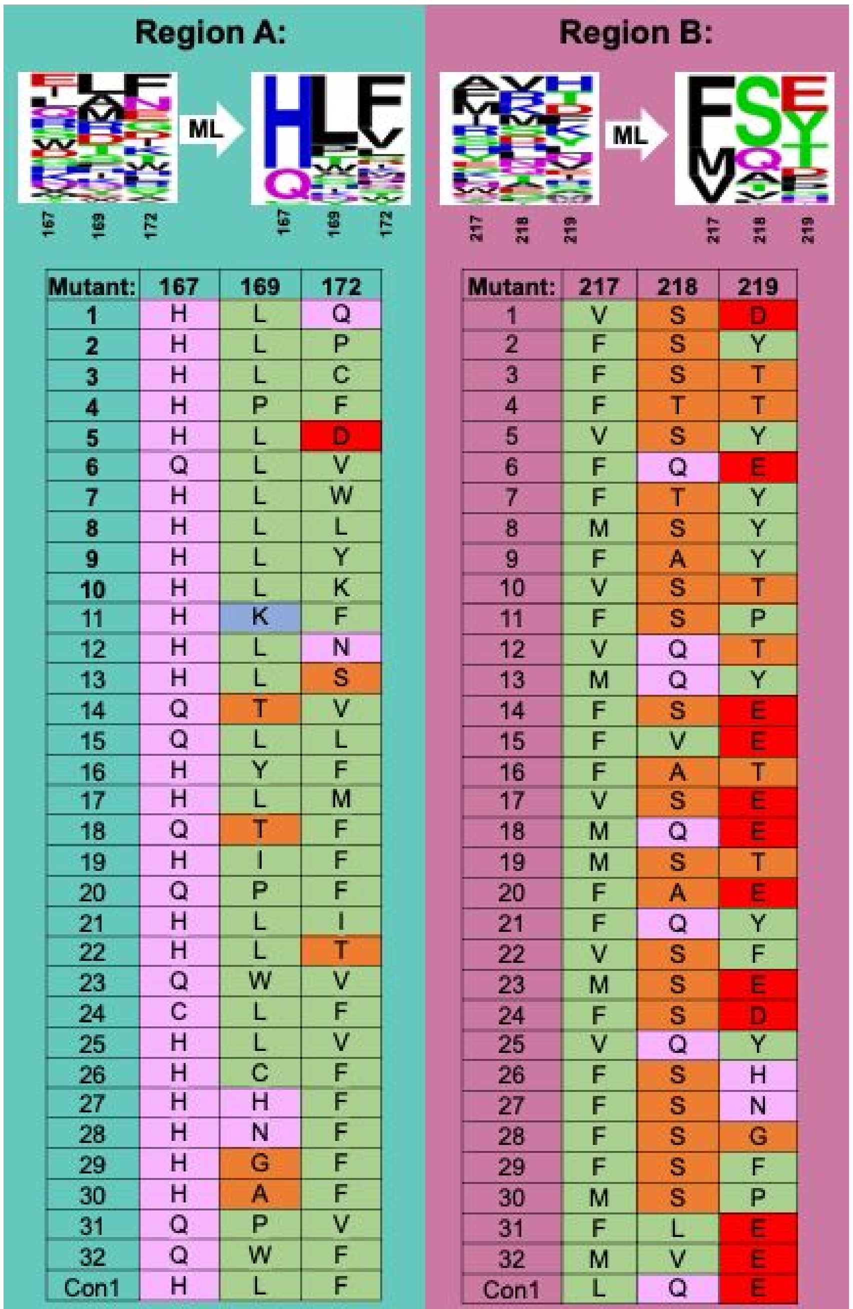

Region A:

Mutant:	167	169	172
1	H	L	Q
2	H	L	P
3	H	L	C
4	H	P	F
5	H	L	D
6	Q	L	V
7	H	L	W
8	H	L	L
9	H	L	Y
10	H	L	K
11	H	K	F
12	H	L	N
13	H	L	S
14	Q	T	V
15	Q	L	L
16	H	Y	F
17	H	L	M
18	Q	T	F
19	H	I	F
20	Q	P	F
21	H	L	I
22	H	L	T
23	Q	W	V
24	C	L	F
25	H	L	V
26	H	C	F
27	H	H	F
28	H	N	F
29	H	G	F
30	H	A	F
31	Q	P	V
32	Q	W	F
Con1	H	L	F

Region B:

Mutant:	217	218	219
1	V	S	D
2	F	S	Y
3	F	S	T
4	F	T	T
5	V	S	Y
6	F	Q	E
7	F	T	Y
8	M	S	Y
9	F	A	Y
10	V	S	T
11	F	S	P
12	V	Q	T
13	M	Q	Y
14	F	S	E
15	F	V	E
16	F	A	T
17	V	S	E
18	M	Q	E
19	M	S	T
20	F	A	E
21	F	Q	Y
22	V	S	F
23	M	S	E
24	F	S	D
25	V	Q	Y
26	F	S	H
27	F	S	N
28	F	S	G
29	F	S	F
30	M	S	P
31	F	L	E
32	M	V	E
Con1	L	Q	E

Figure S7. Identity of mutants suggested by ALDE workflow. Sequence logos of the amino acid identities of the random mutants, compared with machine learning suggested mutants are shown. For region A, only the first 10 of these suggested mutants were tested. For region B, all 32 were screened. The identity of each variant at the residues to be mutated is indicated in the table, with amino acids coloured based on type: green = hydrophobic, pink = polar, red = negatively charged, orange = small non-polar, blue = positively charged.

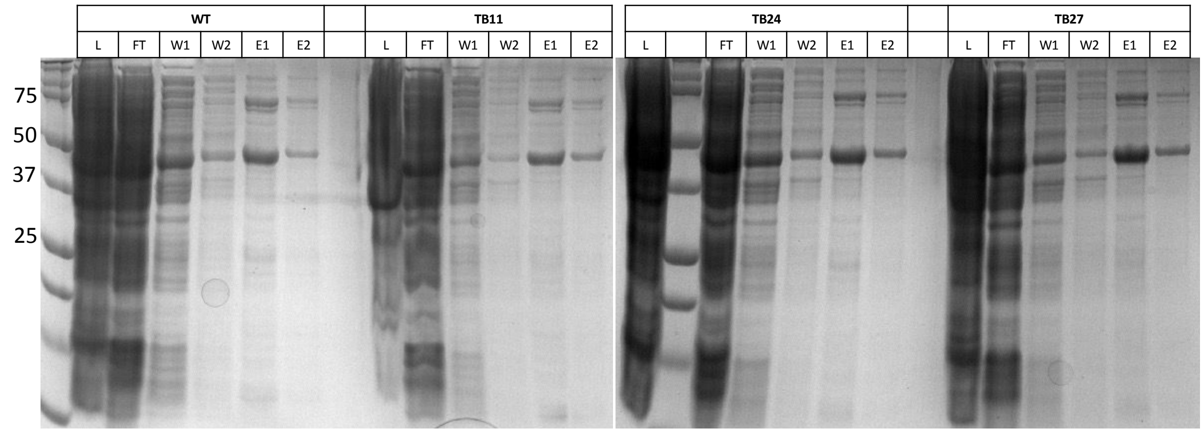

Figure S8. Affinity purification of four protease variants. Indicated by the lane title, different samples from the purification process were loaded: L (lysate), FT (flow-through), W1 (wash 1), W2 (wash 2), E1 (elution 1), E2 (elution 2). Expected MW = 42 kDa.

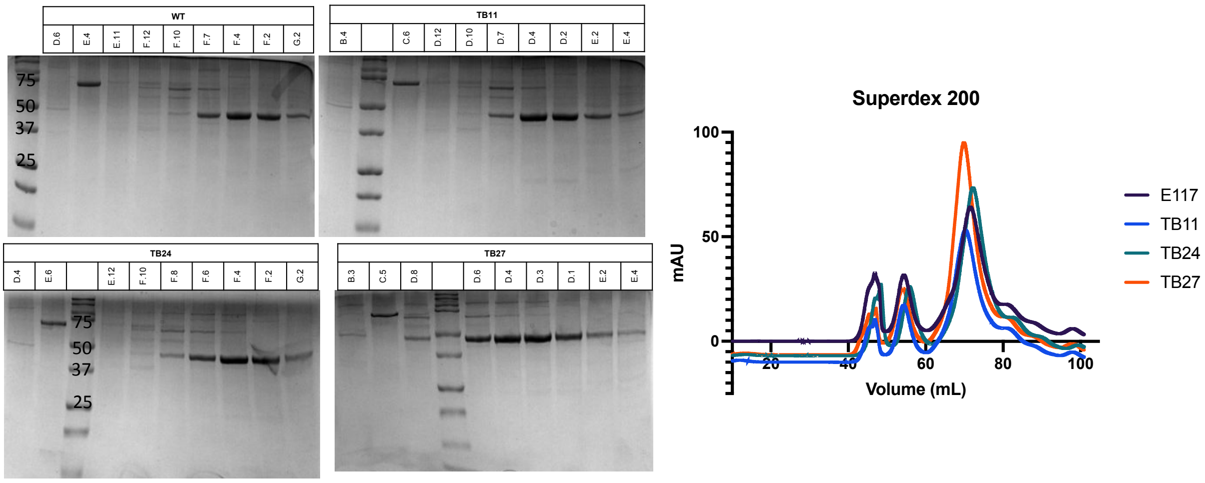

Figure S9. Size exclusion chromatography of four protease variants. Fractions eluted from the column were run on SDS-PAGE to assess purity. Absorbance from the SEC run is shown on the line plot. The largest peak contained the proteases.

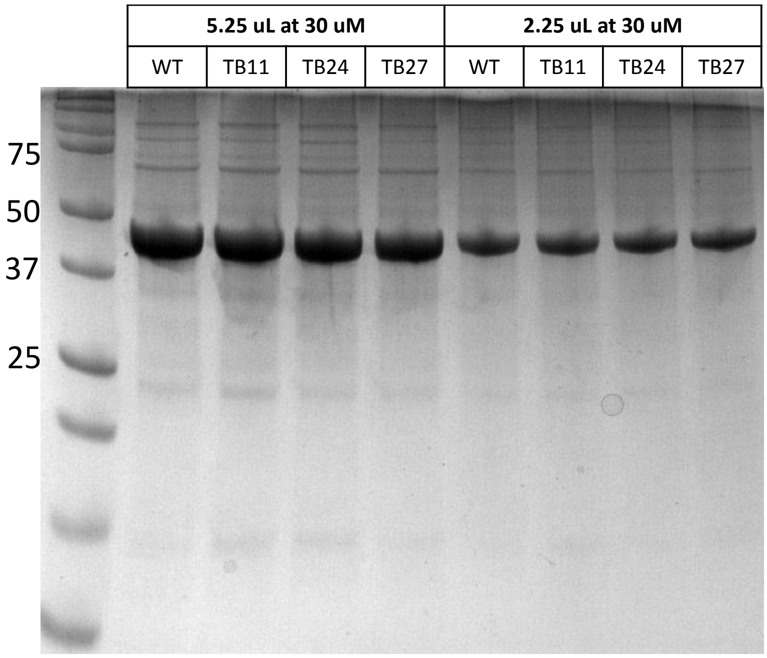

Figure S10. Purified variants stocked at equimolar concentrations.

**Original Con1**	
DNA sequence	ATGCACCATCACCATCACCATGCCGTAACCACCTTATCAGGTTTATCAGGTG<br>AGCAAGGTCCGTCCGGTGATATGACAACTGAAGAAGATAGTGCTACCCATA<br>TTAAATTCTCAAAACGTGATGAGGACGGCCGTGAGTTAGCTGGTGCAACTA<br>TGGAGTTGCGTGATTCATCTGGTAAAACTATTAGTACATGGATTTCAGATGG<br>ACATGTGAAGGATTTCTACCTGTATCCAGGAAAATATACATTTGTCGAAACC<br>GCAGCACCAGACGGTTATGAGGTAGCAACTCCAATTGAATTTACAGTTAATG<br>AGGACGGTCAGGTTACTGTAGATGGTGAAGCAACTGAAGGTGACGCTCATA<br>CTGGAGGTAGCGGTGGCTCAAGCAAATCTTTATTCCGTGGATTACGCGATT<br>ACAACCCGATCGCCAGCAATATTTGTCATTTGACTAATGAGAGCGACGGTC<br>ATAGTAACTCGTTGTATGGCATCGGGTTTGGGCCGCTTATTATTACAAACCA<br>ACATTTGTTTCGTCGCAACAACGGGGAACTTACGATTCAATCTCGCCATGG<br>CGAGTTCGTAGTTAAGAACACCACGCAACTTAAGCTTCTGCCAATCGATGG<br>GCGTGATATCCTTATCATTCGTTTGCCCAAAGATTTCCCACCCTTTCCACAG<br>AAACTTAAATTCCGCCAACCTGAAAAAGGCGAACGCATTTGCTTGGTTGGCT<br>CGAATTTTCAGACCAAGTCCATCACCAGTACAGTCAGCGAAACATCAACAAC<br>CATGCCGGTGGAGAATAGTCAATTTTGGAAGCACTGGATTTCAACCAAAGA<br>CGGTCATTGTGGCCTGCCTCTTGTTTCCACGAAGGATGGCAAGATCCTGGG<br>CATCCATTCTTTGGCGAATTTTACCAATACGATCAACTACTTCGCCGCATTC<br>CCAGAAGATTTTGAGGAGACGTATCTTCATACCCAAGAAGCTCAAGAATGG<br>GTAAAGCACTGGAAATATAACCCGGATGCGATCTCCTGGGGGAGCCTTAAC<br>CTTCAAGAAAGCCAACCAGAAGAGCCTTTCAAAATCGTAAAATTAGTAACGG<br>ACGGAGGTAGCGGTGGCTCAGAACAGAAACTGATTAGCGAAGAAGATCTGT<br>AA
Protein sequence	MHHHHHHAVTTLSGLSGEQGPSGDMTTEEDSATHIKFSKRDEDGRELAGATM<br>ELRDSSGKTISTWISDGHVKDFYLYPGKYTFVETAAPDGYEVATPIEFTVNEDG<br>QVTVDGEATEGDAHTGGSGGSSKSLFRGLRDYNPIASNICHLTNESDGHSNSL<br>YGIGFGPLIITNQHLFRRNNGELTIQSRHGEFVVKNTTQLKLLPIDGRDILIIRLPK<br>DFPPFPQKLKFRQPEKGERICLVGSNFQTKSITSTVSETSTTMPVENSQFWKH<br>WISTKDGHCGLPLVSTKDGKILGIHSLANFTNTINYFAAFPEDFEETYLHTQEAQ<br>EWVKHWKYNPDAISWGSLNLQESQPEEPFKIVKLVTDGGSGGSEQKLISEEDL<br>*
Identity	His-SpyCatcher-Con1-myc

Table S1. Annotated Con1 DNA and protein sequence.

**FRET substrate**	
DNA sequence	ATGCGGGGTTCTCATCATCATCATCATCATGGTATGGCTAGCATGACTGGT<br>GGACAGCAAATGGGTCGGGATCTGTACGACGATGACGATAAGGATCCGGG<br>CCGCATGGTGAGCAAGGGCGAGGAGCTGTTCACCGGGGTGGTGCCCATC<br>CTGGTCGAGCTGGACGGCGACGTAAACGGCCACAAGTTCAGCGTGTCCGG<br>CGAGGGCGAGGGCGATGCCACCTACGGCAAGCTGACCCTGAAGTTCATCT<br>GCACCACCGGCAAGCTGCCCGTGCCCTGGCCCACCCTCGTGACCACCCTG<br>ACCTGGGGCGTGCAGTGCTTCAGCCGCTACCCCGACCACATGAAGCAGCA<br>CGACTTCTTCAAGTCCGCCATGCCCGAAGGCTACGTCCAGGAGCGCACCA<br>TCTTCTTCAAGGACGACGGCAACTACAAGACCCGCGCCGAGGTGAAGTTC<br>GAGGGCGACACCCTGGTGAACCGCATCGAGCTGAAGGGCATCGACTTCAA<br>GGAGGACGGCAACATCCTGGGGCACAAGCTGGAGTACAACTACATCAGCC<br>ACAACGTCTATATCACCGCCGACAAGCAGAAGAACGGCATCAAGGCCAACT<br>TCAAGATCCGCCACAACATCGAGGACGGCAGCGTGCAGCTCGCCGACCAC<br>TACCAGCAGAACACCCCCATCGGCGACGGCCCCGTGCTGCTGCCCGACAA<br>CCACTACCTGAGCACCCAGTCCGCCCTGAGCAAAGACCCCAACGAGAAGC<br>GCGATCACATGGTCCTGCTGGAGTTCGTGACCGCCGCCGGGATCGGTACC<br>GTAGGATTTCTAACAGCGACCGAAGCAGTGTATCATCAATCCCTGGATTCC

	ATCACCGTTAACGGTACCGGTGGAATGGTGAGCAAGGGCGAGGAGCTGTT<br>CACCGGGGTGGTGCCCATCCTGGTCGAGCTGGACGGCGACGTAAACGGC<br>CACAAGTTCAGCGTGTCCGGCGAGGGCGAGGGCGATGCCACCTACGGCAA<br>GCTGACCCTGAAGTTCATCTGCACCACCGGCAAGCTGCCCGTGCCCTGGC<br>CCACCCTCGTGACCACCTTCGGCTACGGCCTGCAGTGCTTCGCCCGCTAC<br>CCCGACCACATGAAGCAGCACGACTTCTTCAAGTCCGCCATGCCCGAAGG<br>CTACGTCCAGGAGCGCACCATCTTCTTCAAGGACGACGGCAACTACAAGAC<br>CCGCGCCGAGGTGAAGTTCGAGGGCGACACCCTGGTGAACCGCATCGAG<br>CTGAAGGGCATCGACTTCAAGGAGGACGGCAACATCCTGGGGCACAAGCT<br>GGAGTACAACTACAACAGCCACAACGTCTATATCATGGCCGACAAGCAGAA<br>GAACGGCATCAAGGTGAACTTCAAGATCCGCCACAACATCGAGGACGGCA<br>GCGTGCAGCTCGCCGACCACTACCAGCAGAACACCCCCATCGGCGACGGC<br>CCCGTGCTGCTGCCCGACAACCACTACCTGAGCTACCAGTCCGCCCTGAG<br>CAAAGACCCCAACGAGAAGCGCGATCACATGGTCCTGCTGGAGTTCGTGA<br>CCGCCGCCGGGATCACTCTCGGCATGGACGAGCTGTACAAGTAA
Protein sequence	MRGSHHHHHHGMASMTGGQQMGRDLYDDDDKDPGRMVSKGEELFTGVVPI<br>LVELDGDVNGHKFSVSGEGEGDATYGKLTLKFICTTGKLPVPWPTLVTTLTWG<br>VQCFSRYPDHMKQHDFFKSAMPEGYVQERTIFFKDDGNYKTRAEVKFEGDTL<br>VNRIELKGIDFKEDGNILGHKLEYNYISHNVYITADKQKNGIKANFKIRHNIEDGS<br>VQLADHYQQNTPIGDGPVLLPDNHYLSTQSALSKDPNEKRDHMVLLEFVTAAGI<br>GTVGFLTATEAVYHQSLDSITVNGTGGMVSKGEELFTGVVPILVELDGDVNGH<br>KFSVSGEGEGDATYGKLTLKFICTTGKLPVPWPTLVTTFGYGLQCFARYPDHM<br>KQHDFFKSAMPEGYVQERTIFFKDDGNYKTRAEVKFEGDTLVNRIELKGIDFKE<br>DGNILGHKLEYNYNSHNVYIMADKQKNGIKVNFKIRHNIEDGSVQLADHYQQNT<br>PIGDGPVLLPDNHYLSYQSALSKDPNEKRDHMVLLEFVTAAGITLGMDELYK*
Identity	His-CFP-cut site-YFP

Table S2. Annotated FRET substrate DNA and protein sequence.

Code	Purpose	Sequence
P291	B_1_F	TTAACCATGTGCTGAGCCAACCAGAAGAGCCTT
P292	B_1_R	GGCTCAGCACATGGTTAAGGCTCCCCCAGGA
P293	A_1_F	GAATCGATTGCCAACAACACCAATACGATCAACTACT
P294	A_1_R	GTTGTTGGCAATCGATTCGATGCCCAGGATCT
P298	B_2_F	TTAACTATCTGATTAGCCAACCAGAAGAGCCTT
P299	B_2_R	GGCTAATCAGATAGTTAAGGCTCCCCCAGGA
P300	B_3_F	TTAACGAAGGCAAAAGCCAACCAGAAGAGCCTT
P301	B_3_R	GGCTTTTGCCTTCGTTAAGGCTCCCCCAGGA
P302	B_4_F	TTAACAACAATCGCAGCCAACCAGAAGAGCCTT
P303	B_4_R	GGCTGCGATTGTTGTTAAGGCTCCCCCAGGA
P304	B_5_F	TTAACCAGACCCATAGCCAACCAGAAGAGCCTT
P305	B_5_R	GGCTATGGGTCTGGTTAAGGCTCCCCCAGGA
P306	B_6_F	TTAACCGCATGCATAGCCAACCAGAAGAGCCTT
P307	B_6_R	GGCTATGCATGCGGTTAAGGCTCCCCCAGGA
P308	B_7_F	TTAACTGGGCGAAAAGCCAACCAGAAGAGCCTT
P309	B_7_R	GGCTTTTCGCCCAGTTAAGGCTCCCCCAGGA
P310	B_8_F	TTAACCATAGCTATAGCCAACCAGAAGAGCCTT
P311	B_8_R	GGCTATAGCTATGGTTAAGGCTCCCCCAGGA
P312	B_9_F	TTAACCATATGTATAGCCAACCAGAAGAGCCTT
P313	B_9_R	GGCTATACATATGGTTAAGGCTCCCCCAGGA
P314	B_10_F	TTAACCTGAACATGAGCCAACCAGAAGAGCCTT
P315	B_10_R	GGCTCATGTTCAGGTTAAGGCTCCCCCAGGA
P317	B_11_R	GGCTGCCCATCGCGTTAAGGCTCCCCCAGGA
P318	B_12_F	TTAACAAATTTTGGAGCCAACCAGAAGAGCCTT
P319	B_12_R	GGCTCCAAAATTTGTTAAGGCTCCCCCAGGA
P320	B_13_F	TTAACTTTCATAGCAGCCAACCAGAAGAGCCTT
P321	B_13_R	GGCTGCTATGAAAGTTAAGGCTCCCCCAGGA
P322	B_14_F	TTAACAGCCATTTTAGCCAACCAGAAGAGCCTT
P323	B_14_R	GGCTAAAATGGCTGTTAAGGCTCCCCCAGGA
P325	B_15_R	GGCTTTCCACCGCGTTAAGGCTCCCCCAGGA
P326	B_16_F	TTAACATGGATCATAGCCAACCAGAAGAGCCTT
P327	B_16_R	GGCTATGATCCATGTTAAGGCTCCCCCAGGA
P328	B_17_F	TTAACATTGTGACCAGCCAACCAGAAGAGCCTT
P329	B_17_R	GGCTGGTCACAATGTTAAGGCTCCCCCAGGA

P330	B_18_F	TTAACAGCAAACAGAGCCAACCAGAAGAGCCTT
P331	B_18_R	GGCTCTGTTTGCTGTTAAGGCTCCCCCAGGA
P332	B_19_F	TTAACGATCATCGCAGCCAACCAGAAGAGCCTT
P333	B_19_R	GGCTGCGATGATCGTTAAGGCTCCCCCAGGA
P334	B_20_F	TTAACCGCAGCAAAAGCCAACCAGAAGAGCCTT
P335	B_20_R	GGCTTTTGCTGCGGTTAAGGCTCCCCCAGGA
P336	B_21_F	TTAACATTGAAGATAGCCAACCAGAAGAGCCTT
P337	B_21_R	GGCTATCTTCAATGTTAAGGCTCCCCCAGGA
P341	B_23_R	GGCTATGGCGCCAGTTAAGGCTCCCCCAGGA
P343	B_24_R	GGCTAAAGCGCGCGTTAAGGCTCCCCCAGGA
P344	B_25_F	TTAACATGGATTATAGCCAACCAGAAGAGCCTT
P345	B_25_R	GGCTATAATCCATGTTAAGGCTCCCCCAGGA
P347	B_26_R	GGCTGCTCACGCTGTTAAGGCTCCCCCAGGA
P348	B_27_F	TTAACGGCTATTATAGCCAACCAGAAGAGCCTT
P349	B_27_R	GGCTATAATAGCCGTTAAGGCTCCCCCAGGA
P350	B_28_F	TTAACGGCAACCATAGCCAACCAGAAGAGCCTT
P351	B_28_R	GGCTATGGTTGCCGTTAAGGCTCCCCCAGGA
P352	B_29_F	TTAACTTTCGCTTTAGCCAACCAGAAGAGCCTT
P353	B_29_R	GGCTAAAGCGAAAGTTAAGGCTCCCCCAGGA
P354	B_30_F	TTAACCGCACCGCGAGCCAACCAGAAGAGCCTT
P355	B_30_R	GGCTCGCGGTGCGGTTAAGGCTCCCCCAGGA
P356	B_31_F	TTAACACCAAAAAGAGCCAACCAGAAGAGCCTT
P357	B_31_R	GGCTCTTTTTGGTGTTAAGGCTCCCCCAGGA
P358	B_32_F	TTAACTTTAGCCTGAGCCAACCAGAAGAGCCTT
P359	B_32_R	GGCTCAGGCTAAAGTTAAGGCTCCCCCAGGA
P360	B_33_F	TTAACCGCAAAAACAGCCAACCAGAAGAGCCTT
P361	B_33_R	GGCTGTTTTTGCGGTTAAGGCTCCCCCAGGA
P362	B_34_F	TTAACATGACCTTCAGCCAACCAGAAGAGCCTT
P363	B_34_R	GGCTGAAGGTCATGTTAAGGCTCCCCCAGGA
P364	B_35_F	TTAACTATGGCGATAGCCAACCAGAAGAGCCTT
P365	B_35_R	GGCTATCGCCATAGTTAAGGCTCCCCCAGGA
P366	B_36_F	TTAACAAAATGATTAGCCAACCAGAAGAGCCTT
P367	B_36_R	GGCTAATCATTTTGTTAAGGCTCCCCCAGGA
P368	B_37_F	TTAACGCGAAAACCAGCCAACCAGAAGAGCCTT
P369	B_37_R	GGCTGGTTTTCGCGTTAAGGCTCCCCCAGGA
P370	B_38_F	TTAACTTCCTGACCAGCCAACCAGAAGAGCCTT
P371	B_38_R	GGCTGGTCAGGAAGTTAAGGCTCCCCCAGGA
P372	B_39_F	TTAACTATGTGGATAGCCAACCAGAAGAGCCTT
P373	B_39_R	GGCTATCCACATAGTTAAGGCTCCCCCAGGA
P374	B_40_F	TTAACTTCCTGGTGAGCCAACCAGAAGAGCCTT
P375	B_40_R	GGCTCACCAGGAAGTTAAGGCTCCCCCAGGA
P376	B_41_F	TTAACATTGAAAACAGCCAACCAGAAGAGCCTT
P377	B_41_R	GGCTGTTTTCAATGTTAAGGCTCCCCCAGGA
P378	B_42_F	TTAACGTGGCGAACAGCCAACCAGAAGAGCCTT
P379	B_42_R	GGCTGTTCGCCACGTTAAGGCTCCCCCAGGA
P380	B_43_F	TTAACATGCAGCATAGCCAACCAGAAGAGCCTT
P381	B_43_R	GGCTATGCTGCATGTTAAGGCTCCCCCAGGA
P382	B_44_F	TTAACATTAGCGATAGCCAACCAGAAGAGCCTT
P383	B_44_R	GGCTATCGCTAATGTTAAGGCTCCCCCAGGA
P384	B_45_F	TTAACGAAGTGACCAGCCAACCAGAAGAGCCTT
P385	B_45_R	GGCTGGTCACTTCGTTAAGGCTCCCCCAGGA
P386	B_46_F	TTAACAACCAGGTGAGCCAACCAGAAGAGCCTT
P387	B_46_R	GGCTCACCTGGTTGTTAAGGCTCCCCCAGGA
P388	B_47_F	TTAACAGCGATACCAGCCAACCAGAAGAGCCTT
P389	B_47_R	GGCTGGTATCGCTGTTAAGGCTCCCCCAGGA
P390	B_48_F	TTAACATGCGCCTGAGCCAACCAGAAGAGCCTT
P391	B_48_R	GGCTCAGGCGCATGTTAAGGCTCCCCCAGGA
P392	A_2_F	ACCTCGCATGCCAACACCACCAATACGATCAACTACT
P393	A_2_R	GGTGTTGGCATGCGAGGTGATGCCCAGGATCT
P394	A_3_F	CGTTCGATGGCCAACTTTACCAATACGATCAACTACT
P395	A_3_R	AAAGTTGGCCATCGAACGGATGCCCAGGATCT
P396	A_4_F	CTGTCGCTGGCCAACGCGACCAATACGATCAACTACT
P397	A_4_R	CGCGTTGGCCAGCGACAGGATGCCCAGGATCT
P398	A_5_F	CGCTCGCGCGCCAACCTGACCAATACGATCAACTACT
P399	A_5_R	CAGGTTGGCGCGCGAGCGGATGCCCAGGATCT
P400	A_6_F	TGGTCGGCGGCCAACAAAACCAATACGATCAACTACT
P401	A_6_R	TTTGTTGGCCGCCGACCAGATGCCCAGGATCT
P402	A_7_F	CGCTCGAAAGCCAACCTGACCAATACGATCAACTACT
P403	A_7_R	CAGGTTGGCTTTCGAGCGGATGCCCAGGATCT
P404	A_8_F	TGGTCGGCGGCCAACTTTACCAATACGATCAACTACT

P405	A_8_R	AAAGTTGGCCGCCGACCAGATGCCCAGGATCT
P406	A_9_F	GGCTCGTTTGCCAACTTTACCAATACGATCAACTACT
P407	A_9_R	AAAGTTGGCAAACGAGCCGATGCCCAGGATCT
P408	A_10_F	CAGTCGCTGGCCAACTTTACCAATACGATCAACTACT
P409	A_10_R	AAAGTTGGCCAGCGACTGGATGCCCAGGATCT
P410	A_11_F	CATTCGACCGCCAACGTGACCAATACGATCAACTACT
P411	A_11_R	CACGTTGGCGGTCGAATGGATGCCCAGGATCT
P412	A_12_F	GGCTCGTATGCCAACCGCACCAATACGATCAACTACT
P413	A_12_R	GCGGTTGGCATACGAGCCGATGCCCAGGATCT
P414	A_13_F	GTGTCGATGGCCAACAACACCAATACGATCAACTACT
P415	A_13_R	GTTGTTGGCCATCGACACGATGCCCAGGATCT
P416	A_14_F	GTGTCGACCGCCAACGATACCAATACGATCAACTACT
P417	A_14_R	ATCGTTGGCGGTCGACACGATGCCCAGGATCT
P418	A_15_F	TATTCGTTTGCCAACAACACCAATACGATCAACTACT
P419	A_15_R	GTTGTTGGCAAACGAATAGATGCCCAGGATCT
P420	A_16_F	TGGTCGCTGGCCAACGGCACCAATACGATCAACTACT
P421	A_16_R	GCCGTTGGCCAGCGACCAGATGCCCAGGATCT
P422	A_17_F	CAGTCGCGCGCCAACGAAACCAATACGATCAACTACT
P423	A_17_R	TTCGTTGGCGCGCGACTGGATGCCCAGGATCT
P424	A_18_F	GAATCGCGCGCCAACTGGACCAATACGATCAACTACT
P425	A_18_R	CCAGTTGGCGCGCGATTCGATGCCCAGGATCT
P426	A_19_F	ATGTCGAGCGCCAACATTACCAATACGATCAACTACT
P427	A_19_R	AATGTTGGCGCTCGACATGATGCCCAGGATCT
P428	A_20_F	GATTCGCTGGCCAACGGCACCAATACGATCAACTACT
P429	A_20_R	GCCGTTGGCCAGCGAATCGATGCCCAGGATCT
P430	A_21_F	GAATCGGATGCCAACTGGACCAATACGATCAACTACT
P431	A_21_R	CCAGTTGGCATCCGATTCGATGCCCAGGATCT
P432	A_22_F	AAATCGAGCGCCAACTTTACCAATACGATCAACTACT
P433	A_22_R	AAAGTTGGCGCTCGATTTGATGCCCAGGATCT
P434	A_23_F	AAATCGATTGCCAACTGGACCAATACGATCAACTACT
P435	A_23_R	CCAGTTGGCAATCGATTTGATGCCCAGGATCT
P438	B_11_F_2	TTAACGCGATGGGCAGCCAACCAGAAGAGCC
P439	B_15_F_2	TTAACGCGGTGGAAAGCCAACCAGAAGAGCC
P440	B_22_F_2	TTAACGCACGTGTGAGCCAACCAGAAGAGCC
P441	B_22_R_2	GGCTCACACGTGCGTTAAGGCTCCCCCAGGA
P442	B_23_F_2	TTAACTGGCGCCATAGCCAACCAGAAGAGCC
P443	B_24_F_2	TTAACGCGCGCTTTAGCCAACCAGAAGAGCC
P444	B_26_F_2	TTAACAGCGTGAGCAGCCAACCAGAAGAGCC
P445	A_24_F_2	GACAGCGCAGCCAACGGCACCAATACGATCAACTACT
P447	A_24_R_2	GCCGTTGGCTGCGCTGTCGATGCCCAGGATCT
P448	A_25_F	GGCTCGAGCGCCAACTATACCAATACGATCAACTACT
P449	A_25_R	ATAGTTGGCGCTCGAGCCGATGCCCAGGATCT
P450	A_26_F	CTGTCGACCGCCAACAAAACCAATACGATCAACTACT
P451	A_26_R	TTTGTTGGCGGTCGACAGGATGCCCAGGATCT
P452	A_27_F	CGTTCGGCGGCCAACCATACCAATACGATCAACTACT
P453	A_27_R	ATGGTTGGCCGCCGAACGGATGCCCAGGATCT
P454	A_28_F	CATTCGATGGCCAACGAAACCAATACGATCAACTACT
P455	A_28_R	TTCGTTGGCCATCGAATGGATGCCCAGGATCT
P456	A_29_F	CAGTCGGTGGCCAACACCACCAATACGATCAACTACT
P457	A_29_R	GGTGTTGGCCACCGACTGGATGCCCAGGATCT
P458	A_30_F	AACTCGAAAGCCAACGATACCAATACGATCAACTACT
P459	A_30_R	ATCGTTGGCTTTCGAGTTGATGCCCAGGATCT
P460	A_31_F	GAATCGCATGCCAACAGCACCAATACGATCAACTACT
P461	A_31_R	GCTGTTGGCATGCGATTCGATGCCCAGGATCT
P462	A_32_F	AGCTCGCGTGCCAACTTTACCAATACGATCAACTACT
P463	A_32_R	AAAGTTGGCACGCGAGCTGATGCCCAGGATCT
P464	A_33_F	TGGTCGCGCGCCAACGAAACCAATACGATCAACTACT
P465	A_33_R	TTCGTTGGCGCGCGACCAGATGCCCAGGATCT
P466	A_34_F	GCGTCGCTGGCCAACTTTACCAATACGATCAACTACT
P467	A_34_R	AAAGTTGGCCAGCGACGCGATGCCCAGGATCT
P468	A_35_F	AGCTCGTGGGCCAACAGCACCAATACGATCAACTACT
P469	A_35_R	GCTGTTGGCCCACGAGCTGATGCCCAGGATCT
P470	A_36_F	ATTTCGGAAGCCAACAACACCAATACGATCAACTACT
P471	A_36_R	GTTGTTGGCTTCCGAAATGATGCCCAGGATCT
P472	A_37_F	ATTTCGCTGGCCAACCATACCAATACGATCAACTACT
P473	A_37_R	ATGGTTGGCCAGCGAAATGATGCCCAGGATCT
P474	A_38_F	CTGTCGATGGCCAACGATACCAATACGATCAACTACT
P475	A_38_R	ATCGTTGGCCATCGACAGGATGCCCAGGATCT
P476	A_39_F	ATTTCGGCGGCCAACAACACCAATACGATCAACTACT
P477	A_39_R	GTTGTTGGCCGCCGAAATGATGCCCAGGATCT

P478	A_40_F	CTGTCGATGGCCAACTTTACCAATACGATCAACTACT
P479	A_40_R	AAAGTTGGCCATCGACAGGATGCCCAGGATCT
P480	A_41_F	ATTTCGTATGCCAACATTACCAATACGATCAACTACT
P481	A_41_R	AATGTTGGCATACGAAATGATGCCCAGGATCT
P482	A_42_F	AGCTCGACCGCCAACGAAACCAATACGATCAACTACT
P483	A_42_R	TTCGTTGGCGGTCGAGCTGATGCCCAGGATCT
P484	A_43_F	GATTCGGATGCCAACACCACCAATACGATCAACTACT
P485	A_43_R	GGTGTTGGCATCCGAATCGATGCCCAGGATCT
P486	A_44_F	GAATCGCAGGCCAACAAAACCAATACGATCAACTACT
P487	A_44_R	TTTGTTGGCCTGCGATTCGATGCCCAGGATCT
P488	A_45_F	AACTCGCAGGCCAACTATACCAATACGATCAACTACT
P489	A_45_R	ATAGTTGGCCTGCGAGTTGATGCCCAGGATCT
P490	A_46_F	AGCTCGGATGCCAACCGCACCAATACGATCAACTACT
P491	A_46_R	GCGGTTGGCATCCGAGCTGATGCCCAGGATCT
P492	A_47_F	CAGTCGCTGGCCAACGGCACCAATACGATCAACTACT
P493	A_47_R	GCCGTTGGCCAGCGACTGGATGCCCAGGATCT
P494	A_48_F	AAATCGGATGCCAACATTACCAATACGATCAACTACT
P495	A_48_R	AATGTTGGCATCCGATTTGATGCCCAGGATCT
P501	TB_1_F	TTAACGTGAGCGACAGCCAACCAGAAGAGCCTT
P502	TB_1_R	GGCTGTCGCTCACGTTAAGGCTCCCCCAGGA
P503	TB_2_F	TTAACTTCAGCTATAGCCAACCAGAAGAGCCTT
P504	TB_2_R	GGCTATAGCTGAAGTTAAGGCTCCCCCAGGA
P505	TB_3_F	TTAACTTCAGCACCAGCCAACCAGAAGAGCCTT
P506	TB_3_R	GGCTGGTGCTGAAGTTAAGGCTCCCCCAGGA
P507	TB_4_F	TTAACTTCACCACGAGCCAACCAGAAGAGCCTT
P508	TB_4_R	GGCTCGTGGTGAAGTTAAGGCTCCCCCAGGA
P509	TB_5_F	TTAACGTGAGCTACAGCCAACCAGAAGAGCCTT
P510	TB_5_R	GGCTGTAGCTCACGTTAAGGCTCCCCCAGGA
P511	TB_6_F	TTAACTTCCAGGAAAGCCAACCAGAAGAGCCTT
P512	TB_6_R	GGCTTTCCTGGAAGTTAAGGCTCCCCCAGGA
P513	TB_7_F	TTAACTTTACCTATAGCCAACCAGAAGAGCCTT
P514	TB_7_R	GGCTATAGGTAAAGTTAAGGCTCCCCCAGGA
P515	TB_8_F	TTAACATGAGCTATAGCCAACCAGAAGAGCCTT
P516	TB_8_R	GGCTATAGCTCATGTTAAGGCTCCCCCAGGA
P517	TB_9_F	TTAACTTCGCATATAGCCAACCAGAAGAGCCTT
P518	TB_9_R	GGCTATATGCGAAGTTAAGGCTCCCCCAGGA
P519	TB_10_F	TTAACGTTAGCACCAGCCAACCAGAAGAGCCTT
P520	TB_10_R	GGCTGGTGCTAACGTTAAGGCTCCCCCAGGA
P521	TB_11_F	TTAACTTCAGCCCGAGCCAACCAGAAGAGCCTT
P522	TB_11_R	GGCTCGGGCTGAAGTTAAGGCTCCCCCAGGA
P523	TB_12_F	TTAACGTGCAGACCAGCCAACCAGAAGAGCCTT
P524	TB_12_R	GGCTGGTCTGCACGTTAAGGCTCCCCCAGGA
P525	TB_13_F	TTAACATGCAGTATAGCCAACCAGAAGAGCCTT
P526	TB_13_R	GGCTATACTGCATGTTAAGGCTCCCCCAGGA
P527	TB_14_F	TTAACTTTAGCGAAAGCCAACCAGAAGAGCCTT
P528	TB_14_R	GGCTTTCGCTAAAGTTAAGGCTCCCCCAGGA
P529	TB_15_F	TTAACTTTGTGGAAAGCCAACCAGAAGAGCCTT
P530	TB_15_R	GGCTTTCCACAAAGTTAAGGCTCCCCCAGGA
P531	TB_16_F	TTAACTTTGCAACCAGCCAACCAGAAGAGCCTT
P532	TB_16_R	GGCTGGTTGCAAAGTTAAGGCTCCCCCAGGA
P533	TB_17_F	TTAACGTGAGCGAAAGCCAACCAGAAGAGCCTT
P534	TB_17_R	GGCTTTCGCTCACGTTAAGGCTCCCCCAGGA
P535	TB_18_F	TTAACATGCAGGAAAGCCAACCAGAAGAGCCTT
P536	TB_18_R	GGCTTTCCTGCATGTTAAGGCTCCCCCAGGA
P537	TB_19_F	TTAACATGAGCACGAGCCAACCAGAAGAGCCTT
P538	TB_19_R	GGCTCGTGCTCATGTTAAGGCTCCCCCAGGA
P539	TB_20_F	TTAACTTCGCAGAAAGCCAACCAGAAGAGCCTT
P540	TB_20_R	GGCTTTCTGCGAAGTTAAGGCTCCCCCAGGA
P541	TB_21_F	TTAACTTCCAGTATAGCCAACCAGAAGAGCCTT
P542	TB_21_R	GGCTATACTGGAAGTTAAGGCTCCCCCAGGA
P543	TB_22_F	TTAACGTGAGCTTTAGCCAACCAGAAGAGCCTT
P544	TB_22_R	GGCTAAAGCTCACGTTAAGGCTCCCCCAGGA
P545	TB_23_F	TTAACATGAGCGAAAGCCAACCAGAAGAGCCTT
P546	TB_23_R	GGCTTTCGCTCATGTTAAGGCTCCCCCAGGA
P547	TB_24_F	TTAACTTTAGCGATAGCCAACCAGAAGAGCCTT
P548	TB_24_R	GGCTATCGCTAAAGTTAAGGCTCCCCCAGGA
P549	TB_25_F	TTAACGTGCAGTATAGCCAACCAGAAGAGCCTT
P550	TB_25_R	GGCTATACTGCACGTTAAGGCTCCCCCAGGA
P551	TB_26_F	TTAACTTTAGCCATAGCCAACCAGAAGAGCCTT
P552	TB_26_R	GGCTATGGCTAAAGTTAAGGCTCCCCCAGGA

P553	TB_27_F	TTAACTTTAGCAACAGCCAACCAGAAGAGCCTT
P554	TB_27_R	GGCTGTTGCTAAAGTTAAGGCTCCCCCAGGA
P555	TB_28_F	TTAACTTTAGCGGCAGCCAACCAGAAGAGCCTT
P556	TB_28_R	GGCTGCCGCTAAAGTTAAGGCTCCCCCAGGA
P557	TB_29_F	TTAACTTCAGCTTTAGCCAACCAGAAGAGCCTT
P558	TB_29_R	GGCTAAAGCTGAAGTTAAGGCTCCCCCAGGA
P559	TB_30_F	TTAACATGAGCCCGAGCCAACCAGAAGAGCCTT
P560	TB_30_R	GGCTCGGGCTCATGTTAAGGCTCCCCCAGGA
P561	TB_31_F	TTAACTTTCTGGAAAGCCAACCAGAAGAGCCTT
P562	TB_31_R	GGCTTTCCAGAAAGTTAAGGCTCCCCCAGGA
P563	TB_32_F	TTAACATGGTGGAAAGCCAACCAGAAGAGCCTT
P564	TB_32_R	GGCTTTCCACCATGTTAAGGCTCCCCCAGGA
P565	TA_1_F	CATTCGCTGGCCAACCAGACCAATACGATCAACTACT
P566	TA_1_R	CTGGTTGGCCAGCGAATGGATGCCCAGGATCT
P567	TA_2_F	CATTCGCTGGCCAACCCGACCAATACGATCAACTACT
P568	TA_2_R	CGGGTTGGCCAGCGAATGGATGCCCAGGATCT
P569	TA_3_F	CATTCGCTGGCCAACTGCACCAATACGATCAACTACT
P570	TA_3_R	GCAGTTGGCCAGCGAATGGATGCCCAGGATCT
P571	TA_4_F	CATTCGCCGGCCAACTTCACCAATACGATCAACTACT
P572	TA_4_R	GAAGTTGGCCGGCGAATGGATGCCCAGGATCT
P573	TA_5_F	CATTCGCTGGCCAACGATACCAATACGATCAACTACT
P574	TA_5_R	ATCGTTGGCCAGCGAATGGATGCCCAGGATCT
P575	TA_6_F	CAGTCGCTGGCCAACGTGACCAATACGATCAACTACT
P576	TA_6_R	CACGTTGGCCAGCGACTGGATGCCCAGGATCT
P577	TA_7_F	CATTCGCTGGCCAACTGGACCAATACGATCAACTACT
P578	TA_7_R	CCAGTTGGCCAGCGAATGGATGCCCAGGATCT
P579	TA_8_F	CATTCGCTGGCCAACCTGACCAATACGATCAACTACT
P580	TA_8_R	CAGGTTGGCCAGCGAATGGATGCCCAGGATCT
P581	TA_9_F	CATTCGCTGGCCAACTATACCAATACGATCAACTACT
P582	TA_9_R	ATAGTTGGCCAGCGAATGGATGCCCAGGATCT
P583	TA_10_F	CATTCGCTGGCCAACAAAACCAATACGATCAACTACT
P584	TA_10_R	TTTGTTGGCCAGCGAATGGATGCCCAGGATCT

Table S3. Oligonucleotides used in this study.
